# Prevalence of cannabidiol use and correlates in U.S. adults

**DOI:** 10.1016/j.dadr.2024.100289

**Published:** 2024-10-09

**Authors:** Namkee G. Choi, C. Nathan Marti, Bryan Y. Choi

**Affiliations:** a Steve Hicks School of Social Work, University of Texas at Austin, USA; b Department of Emergency Medicine, Philadelphia College of Osteopathic Medicine and BayHealth, USA

**Keywords:** CBD, Cannabis, Chronic medical condition, Mental illness, Cannabis risk perception

## Abstract

**Background:**

Cannabidiol (CBD) use has been increasing for its putative therapeutic potential for various health conditions. Research using a nationally representative sample is needed to examine characteristics of CBD users.

**Methods:**

Data came from the adult sample (N=47,100) of the 2022 U.S. National Survey on Drug Use and Health. We fitted generalized linear models to examine the sociodemographic, health, other substance use, and cannabis risk perception as correlates of CBD-only use and CBD-cannabis co-use, compared to cannabis-only use.

**Results:**

In 2022, 20.6 % and 23.0 % of U.S. adults reported using CBD and cannabis, respectively, in the preceding 12 months, and 63 % of CBD users also used cannabis. CBD use was significantly higher among women (CBD-only vs. cannabis-only use: IRR=1.43, 95 % CI=1.31–1.57), but significantly lower among Black and Hispanic individuals compared to non-Hispanic White individuals (CBD-only vs. cannabis-only use: IRR=0.71, 95 % CI=0.60–0.85 for Black individuals; IRR=0.79, 95 % CI=0.65–0.96 for Hispanic individuals). Older ages, higher SES, chronic medical conditions, mental illness, and high cannabis risk perception were also associated with higher likelihood of CBD-only use versus cannabis-only use. CBD-cannabis co-users were at most risk in terms of chronic illness, mental illness, cannabis use disorder, and other substance use problems.

**Conclusions:**

The high prevalence of self-reported CBD use among those with physical and mental health problems warrants public health warnings about potential side effects and drug interactions. The high CBD-cannabis co-use rate also calls for more research on potential benefits and negative effects of the co-use.

## Highlights

• In 2022, 20 % of U.S. adults reported past-year CBD or hemp-derived product use.
• Almost two thirds of CBD or hemp product users also used cannabis.
• Women were more likely to use CBD or hemp products, with or without cannabis co-use.
• Higher SES, chronic illness, and mental illness were also CBD and hemp product-use correlates.
• More CBD/hemp product-cannabis co-users than cannabis-only users had cannabis use disorder.

## 1 Introduction

Cannabidiol or CBD can be derived from hemp (any part of the *Cannabis sativa* plant with no more than 0.3 % of tetrahydrocannabinol or THC) or manufactured in a laboratory. Unlike psychoactive THC/cannabis consumption, acute or short-term CBD consumption has been found to have no significant adverse effects impairing daily functioning or risks of addiction, although subjective ratings of sedation, drowsiness, or some cognitive delays in healthy individuals were noted as side effects (Lo et al., 2024, Manning et al., 2024). Since the 2018 Farm Bill legalized industrial hemp with THC concentration of <0.3 %, CBD-containing products, including oils, tinctures, gummies, topicals, and pills/capsules, are widely available at pharmacies, cannabis dispensaries, and online marketplaces, and websites. CBD users have been drawn to it seeking relief from chronic pain, insomnia, anxiety, depression, stress, and various other health problems, and most of them report high perceived health and wellness benefits (Corroon and Phillips, 2018, Fedorova et al., 2021, Geppert et al., 2023, Goodman et al., 2022, Kaufmann et al., 2023, Leas et al., 2020, Moltke and Hindocha, 2021, Binkowska et al., 2024).

CBD’s promising therapeutic efficacy and safety have been established in pediatric patients with Lennox-Gastaut syndrome or Dravet syndrome, which are rare and severe forms of epilepsy with onset in early childhood (Lattanzi et al., 2019, CBD EAP study group., 2019, Aderinto et al., 2024). Epidiolex for Lennox-Gastaut and Dravet syndromes is the only CBD-based prescription medicine approved by the U.S. Food and Drug Administration (FDA). Despite the popular beliefs about its therapeutic benefits, scientific clinical research for CBD’s other health benefits is at its incipient stage. Meta-analyses and systematic reviews thus far found little or uncertain positive medicinal effects of CBD for chronic neuropathic pain (Mücke et al., 2018), chronic neurological pain in multiple sclerosis (Filippini et al., 2022), pain in people with advanced cancer (Häuser et al., 2023), and neurodevelopmental disorders (Parrella et al., 2023). In a clinical trial for Parkinson’s disease, no benefit was reported, with possible worsened cognition and sleep, and many mild adverse events (Liu et al., 2024).

CBD appears to be more promising for psychiatric conditions. Meta-analyses found that CBD can ameliorate anxiety, psychosis, substance use disorders, and PTSD, but more controlled studies and clinical trials, particularly investigating the mid- to long-term use of CBD, are required to conclusively establish its efficacy and safety in treating these conditions (Dammann et al., 2024, Khan et al., 2020, Kirkland et al., 2022). CBD might also be efficacious as a treatment for cannabis use disorder (CUD) (Bhardwaj et al., 2024) and nicotine and opioid use disorders (Paulus et al., 2022). A systematic review found that CBD may reduce drug cravings and other addiction-related symptoms and may have utility as an adjunct harm reduction strategy for people who use drugs (Lo et al., 2023). For most CBD products, however, the effectiveness and the level of appropriate concentrations and dosages, potential interactions with other drugs, and long-term safety are unknown (Kolli and Hoeng, 2024, Revol et al., 2024). Furthermore, studies found that most commercially available CBD products did not meet the definition of the product type claimed on the packaging and deviated from their label claim of CBD potency, contained THC, and showed lot-to-lot variability, making dosing unpredictable (Gidal et al., 2024, Miller et al., 2022, Spindle et al., 2022).

CBD’s popularity among the public has been shown in the 2019 International Cannabis Policy Study (ICPS) that was conducted in the United States and Canada via web-based surveys with respondents ages 16–65 years. The study showed that 26 % of 30,288 U.S. respondents and 16 % of 15,042 Canadian respondents reported past-year CBD use (Goodman et al., 2022). The U.S. CBD users were more likely to be women and white, and 16 % of the users used it daily/almost daily (Goodman et al., 2022). This and other studies have also shown that CBD use is significantly higher among those who also use cannabis and other drugs (Corroon and Phillips, 2018, Dunbar et al., 2022, Goodman et al., 2022, Wheeler et al., 2020; Vilches et al., 2021). In Dunbar et al.’s study (2022) of young adults in California in 2019–2020, 79 % of past-month CBD users (13 % of the survey respondents) were also past-month cannabis users.

Previous studies of CBD users were informative; however, they were done with small clinical samples focusing on specific health conditions or self-selected convenience samples who tended to be young adults. Given the high prevalence of pain and sleep problems among older adults, they are likely to be an increasing share of CBD users and need to be included in CBD research. Another knowledge gap is related to the potential differences among different groups of CBD users, such as CBD-only users (i.e., CBD use without cannabis use) and CBD-cannabis co-users, with respect to their sociodemographic, physical and mental health, and other substance use characteristics.

In the present study based on the 2022 National Survey on Drug Use and Health (NSDUH) that included the questions about past-year and past-month use of “CBD or hemp products made from hemp plants” (referred to as CBD hereafter for brevity) for the first time in the NSDUH history, we first examined the CBD use prevalence among U.S. adults and compared CBD-only users, cannabis-only users (i.e., cannabis use without CBD use), and CBD-cannabis co-users to those of non-users of CBD or cannabis with respect to sociodemographic and clinical characteristics and cannabis risk perception. Then, we examined the correlates of CBD-only use versus cannabis only use and the correlates of CBD-cannabis co-use versus cannabis-only use. The study hypotheses were: (H1) compared to non-users of CBD or cannabis, CBD-only users would be female and non-Hispanic white, have more physical and mental health problems, and have lower cannabis risk perception; (H2) compared to cannabis-only users, CBD-only users would be older, female, and non-Hispanic white, have higher socio-economic status, have more chronic conditions, and have higher cannabis risk perception, but would be less likely to have other substance use problems; and (H3) compared to cannabis-only users, CBD-cannabis co-users would be younger and more likely to have mental illnesses, other substance use problems, and CUD. The findings based on the nationally representative sample will expand the knowledge base of CBD users and help better understand their characteristics.

## 2 Materials and methods

### 2.1 Data source

The NSDUH is an annual epidemiologic survey, funded by the U.S. Substance Abuse and Mental Health Services Administration (SAMHSA), of the civilian, non-institutionalized, age 12+ population to measure the prevalence of substance use, mental and substance use disorders, behavioral health treatment, and physical/functional health and healthcare use. Because of the ongoing COVID-19 pandemic, data collection in 2022 was done using both in-person and web-based modes, with 40.7 % of data collected via the web (Center for Behavioral Health Statistics and Quality, 2023). The 2022 NSDUH public use data set includes responses from a total of 59,069 individuals who completed an in-person or web-based NSDUH survey and imputed values for missing data. In this study, we focused on 47,100 adults age 18 years or older. Analysis of these de-identified public-use data was exempt from the authors’ institutional review boards’ review.

### 2.2 Measures

#### 2.2.1 CBD and cannabis use

In the 2022 NSDUH, respondents were asked two questions about “CBD or hemp products made from hemp plants”: (1) Ever use, even once, of any CBD or hemp products; and (2) time since the last use of any form of CBD or hemp product. Respondents were also asked about use and time since the last use of “marijuana or any cannabis product, sometimes called pot, weed, hashish, or concentrates, excluding CBD or hemp products.” Based on the responses to these questions, we created the past-year and past month use variables with the attributes of non-use of CBD or cannabis, CBD-only use, cannabis-only use, and CBD-cannabis co-use.

#### 2.2.2 Cannabis use-related characteristics of past-year cannabis users

included the first age of use, number of days used (1–11, 12–49, 50–99, 100–299, and 300–365), medical use, and *DSM-5* CUD and CUD severity (mild [2–3 symptoms], moderate [4–5 symptoms], or sever [6–11 symptoms]) (American Psychiatric Association., 2013).

*2.2.3. Cannabis risk perception*: All NSDUH respondents were asked a question, “*How much do people risk harming themselves physically and in other ways when they smoke marijuana once or twice a week*?” The response categories were no risk, slight risk, moderate risk, and great risk. In this study, we used three categories (no/slight risk, moderate risk, and great risk) in bivariate analyses, but used a dichotomous category (great risk vs. no great risk) in multivariable analyses. Great risk perceptions for smoking 1+ pack of cigarette a day and binge alcohol use 1–2 times a week were provided for comparative purposes only.

#### 2.2.3 Sociodemographic factors

were age (18–34, 35–49, 50–64, and 65+ years); gender; race/ethnicity; education (college degree vs. no college degree); income (<poverty line, up to 2X poverty line, >2X poverty line); geographic area of residence (large metropolitan area, small metropolitan area, non-metropolitan area); and the residence in a medical cannabis legal state (yes vs. no). Marital status was reported for descriptive purposes only.

#### 2.2.4 Diagnosed chronic illness, past-year mental illness, and other substance use problems

The number (0−10) of diagnosed chronic illnesses included asthma, cancer, COPD, diabetes, heart disease, hepatitis, HIV/AIDS, hypertension, kidney disease, and liver disease. Any past-year mental illness was categorized as none, mild, moderate, serious (any mental, behavioral, or emotional disorder--excluding developmental and substance use disorders--that substantially interfered with or limited one or more major life activities; SAMHSA, 2020). Past-year other substance use problems included in the study were nicotine dependence (Heatherton, et al., 1991), *DSM-5* alcohol use disorder, and the use of any illicit drugs excluding cannabis.

### 2.3 Analysis

We used Stata/MP 18’s svy function (College Station, TX) and subpop command in all analyses to account for NSDUH’s multi-stage, stratified sampling estimates to ensure that variance estimates incorporate the full sampling design. All estimates presented in this study are weighted except sample sizes. First, we calculated the prevalence, with 95 % confidence intervals (CI), of past-year and past-month CBD and cannabis use in the U.S. adult population. Second, we used Pearson’s χ^2^ tests, ANOVA, and t tests to compare sociodemographic, health, and other substance use characteristics, and cannabis and other substance use risk perceptions among non-users of CBD or cannabis, CBD-only users, cannabis-only users, and CBD-cannabis co-users. We also compared cannabis use-related characteristics between cannabis-only users and CBD-cannabis co-users. Third, we fit three generalized linear models (GLM) for a Poisson distribution with a log link function to test H1 (correlates of CBD-only use vs. non-use of CBD or cannabis), H2 (correlates of CBD-only use vs. cannabis-only use), and H3 (CBD-cannabis co-use vs. cannabis-only use). GLM model results are presented as incidence rate ratios (IRR) with 95 % CI. We used the Poisson distribution with a log link rather than logistic regression models because odds ratios exaggerate the true relative risk to some degree when the event (i.e., CBD use) is a common (i.e., >10 %) occurrence (Grimes & Schulz, 2008). As a preliminary diagnostic, variance inflation factor (VIF), using a cut-off of 2.50 (Allison, 2012), from linear regression models was used to assess multicollinearity among covariates. VIF diagnostics indicated that multicollinearity was not a concern. Significance was set at *p<*.05.

## 3 Results

### 3.1 CBD and cannabis use prevalence

Table 1 shows that 20.6 % and 23.0 % of the U.S. adult population reported using CBD and cannabis, respectively, in the past 12 months; 7.7 % reported CBD-only use, 10.1 % reported cannabis-only use, and 12.9 % reported CBD-cannabis co-use, indicating that 62.5 % of CBD users also used cannabis and 56.0 % of cannabis-users also used CBD. In the preceding month, 11.0 % and 16.0 % reported using CBD and cannabis, respectively. Additional analyses showed that CBD-only use was the highest in the 65+ age group (9.9 % vs. 5.6 %, 7.1 %, and 8.9 % in the 18–34, 35–49, and 50–64 age groups, respectively), whereas cannabis-only use was highest in the 18–34 age group (16.2 % vs. 10.1 %, 8.7 %, and 3.6 % in the 35–49, 50–64, and 65+ age groups, respectively) (F [6.86, 342.85]=78.27, p<.001).

**Table 1:** Cannabis and CBD use among U.S. adults in 2022.

		% of the adult population	95 % confidence interval
**Past year**			
CBD		20.6	19.8–21.4
Cannabis		23.0	22.2–23.7
CBD and/or cannabis use			
	No use	69.3	68.4–70.2
	CBD only	7.7	7.2–8.2
	Cannabis only	10.1	9.6–10.6
	Both CBD and cannabis	12.9	12.2–13.5
**Past month**			
	CBD	11.0	10.4–11.6
	Cannabis	16.0	15.4–16.6
CBD and/or cannabis use			
	No use	79.5	78.8–80.2
	CBD only	4.5	4.1–5.0
	Cannabis only	9.5	9.1–9.9
	Both CBD and cannabis	6.5	6.1–6.9

### 3.2 Characteristics of past-year CBD and cannabis users

Table 2 shows that compared to non-users of CBD or cannabis, CBD-only users included higher proportions of females, non-Hispanic whites, married/partnered individuals, higher SES (in terms of education and income) people, and those with chronic illness, mental illness and substance use problems. CBD-only users had lower risk perceptions of binge alcohol use and smoking cannabis.

**Table 2:** Characteristics of past-year cannabis and CBD users compared to non-users.

		Non-use of cannabis or CBD (a) 30,944 (69.3 %)	CBD only (b) 3360 (7.7 %)	Cannabis only (c) 5677 (10.1 %)	Both CBD & cannabis (d) 7119 (12.9 %)	*P-value:* (a) vs. (b)	*P-value:* (b) vs. (c)	*P-value:* (c) vs. (d)
Age (%, years)						1.00	<.001	.158
	18–34	24.2	21.2	46.8	47.7			
	35–49	24.1	22.5	24.5	27.1			
	50–64	25.5	27.9	20.8	17.6			
	65+	26.2	28.5	8.0	7.6			
Female (%)		53.1	61.1	39.9	49.2	<.001	<.001	<.001
Race/ethnicity (%)						<.001	<.001	<.001
	Non-Hispanic white	59.5	74.7	60.1	67.3			
	Non-Hispanic Black	12.3	8.2	15.9	10.1			
	Hispanic	18.5	9.4	16.7	15.7			
	Asian/Pacific Islander	7.7	4.9	3.9	3.1			
	American Indian/Alaska Native	0.4	0.5	0.7	0.6			
	Multiracial/Other	1.5	2.3	2.6	3.2			
Marital status						.001	<.001	.445
	Married/partnered	52.5	58.7	33.3	34.7			
	Widowed	6.7	6.3	2.6	1.9			
	Divorced/separated	13.8	14.5	14.3	15.2			
	Never married	27.1	20.6	49.8	48.2			
College degree (%)		33.7	39.5	29.0	30.4	<.001	<.001	.420
Income (%)						<.001	<.001	.382
	Below poverty line	19.5	15.2	20.4	20.2			
	Up to 2X poverty line	13.9	9.4	17.6	15.8			
	More than 2X poverty line	66.6	75.4	62.0	64.1			
Geographic area (%)						.724	.021	.025
	Large metropolitan area	54.7	53.6	59.2	54.4			
	Small metropolitan area	31.6	32.7	29.7	34.0			
	Non-metropolitan area	13.7	13.7	11.1	11.6			
Residence in medical cannabis legal states (%)		71.5	70.6	78.6	78.2	.613	<.001	.735
Chronic illness (M, SE)		0.69 (0.01)	0.90 (0.03)	0.49 (0.03)	0.59 (0.02)	<.001	<.001	.013
Any mental illness (%)						<.001	.009	<.001
	None	82.9	71.6	65.7	56.5			
	Mild	8.9	12.8	14.0	15.4			
	Moderate	4.6	7.5	9.7	14.0			
	Severe	3.6	8.1	10.7	14.1			
Serious suicidal thoughts (%)		3.3	4.9	9.4	12.8	.003	<.001	<.001
Nicotine dependence (%)		5.9	5.0	16.0	13.6	.210	<.001	.027
Alcohol use disorder (%)		6.9	10.2	24.6	25.1	<.001	<.001	.672
Illicit drug use excluding cannabis (%)		3.5	6.9	22.3	30.3	<.001	<.001	<.001
Substance use disorder excluding cannabis use disorder (%)		2.4	3.7	8.1	10.5	.015	<.001	.015
Great risk for smoking 1+ pack of cigarette a day (%)		70.3	70.7	61.0	64.1	.776	<.001	.061
Great risk perception for binge alcohol use 1–2 times a week (%)		46.7	40.8	32.7	33.4	<.001	<.001	.667
Risk perception for smoking cannabis 1–2 times weekly (%)						<.001	<.001	.018
	No or slight risk	42.0	59.2	86.7	88.8			
	Moderate risk	25.9	23.9	10.8	8.0			
	Great risk	32.1	16.9	2.5	3.1			

Note: *P*-values were calculated based on Pearson’s *χ*^2^ tests and *t* tests.

Compared to cannabis-only users, CBD-only users included higher proportions of the 65+ age group, females, non-Hispanic whites, married/partnered individuals, higher SES people, and those with chronic illnesses but lower proportions of people with any mental illness and substance use problems. They also had higher risk perceptions of cigarette smoking, binge alcohol use, and cannabis smoking.

Compared to cannabis-only users, CBD-cannabis co-users included higher proportions of females, non-Hispanic whites, those residing in small metropolitan areas, and those with chronic illness, mental illness and substance use problems. Only small proportions of cannabis-only users (2.5 %) and CBD-cannabis co-users (3.1 %) perceived a great risk perception for smoking cannabis 1–2 times a week.

Additional analyses showed that in each of four groups (i.e., non-use of CBD or cannabis, CBD-only use, cannabis-only use, and CBD-cannabis co-use), the numbers of chronic illnesses were significantly higher but the proportion with any mental illness was significantly lower in older age groups. For example, among CBD-only users, the 18–34 age group and the 65+ age group had an average of 0.34 (SE=0.03) and 1.35 (SE=0.07), respectively, chronic illnesses (p<.001), and 47.5 % of the 18–34 age group and 12.0 % of the 65+ age group had any mental illness (p<.001).

### 3.3 Cannabis use related characteristics

Table 3 shows that compared to cannabis-only users, CBD-cannabis co-users included higher proportions of medical cannabis users, 100+ day users, and those with moderate or severe CUD.

**Table 3:** Cannabis and CBD use characteristics by past-year CBD-cannabis use status.

		Cannabis only 5677 (44.0 %)	Both CBD & cannabis 7119 (56.0 %)	*P*-values
First age of cannabis use <18 years (%)		56.9	58.7	.263
Any medical cannabis use (%)		13.1	19.9	<.001
All medical cannabis use (%)		8.7	12.9	<.001
Cannabis use days (%)				<.001
	1–11 days	29.7	20.7	
	12–49 days	16.0	16.3	
	50–99 days	9.9	9.9	
	100–299 days	21.6	25.7	
	300+ days	22.7	27.4	
Cannabis use disorder (CUD; %)				.005
	No CUD	72.4	67.5	
	Mild CUD	16.0	17.6	
	Moderate CUD	7.3	9.3	
	Severe CUD	4.3	5.6	

Note: *P*-values were calculated based on Pearson’s *χ*^2^ tests.

### 3.4 Correlates of CBD-only use versus no CBD or cannabis use

The second column of Table 4 shows that compared to non-use of CBD or cannabis, the likelihood of CBD-only use was significantly associated with female gender (IRR=1.42, 95 % CI=1.22–1.64), chronic illness (IRR=1.11, 95 % CI=1.05–1.18), mental illness (IRR=1.87, 95 % CI=1.57–2.24 for severe illness), alcohol use disorder (IRR=1.27, 95 % CI=1.04–1.54), and illicit drug use (IRR=1.62, 95 % CI=1.25–2.10). Being non-Hispanic Black (IRR=0.63, 95 % CI=0.49–0.80) and being Hispanic (IRR=0.54, 95 % CI=0.44–0.67) compared to being non-Hispanic White, income above the poverty line but no more than 2X poverty line, nicotine dependence, and great cannabis risk perception were associated with a lower likelihood of CBD-only use.

**Table 4:** Correlates of CBD-only use versus no CBD or cannabis use and correlates of CBD-only and both cannabis and CBD use versus cannabis-only use: Generalized linear modeling results.

			CBD-only use vs. No CBD or cannabis use IRR (95 % CI)	CBD-only use vs. Cannabis-only use IRR (95 % CI)	Both cannabis and CBD use vs. Cannabis-only use IRR (95 % CI)
Age: vs. 18–34 years					
	35–49		1.09 (0.95–1.26)	1.45 (1.28–1.65)***	1.05 (0.99–1.11)
	50–64		1.22 (0.99–1.49)	1.60 (1.40–1.82)***	0.93 (0.83–1.05)
	65+		1.21 (0.99–1.47)	1.94 (1.70–2.22)***	0.96 (0.84–1.10)
Female vs. Male			1.42 (1.22–1.64)***	1.43 (1.31–1.57)***	1.17 (1.10–1.25)***
Race/ethnicity: vs. Non-Hispanic white					
	Non-Hispanic Black		0.63 (0.49–0.80)***	0.71 (0.60–0.85)***	0.79 (0.72–0.86)***
	Hispanic		0.54 (0.44–0.67)***	0.79 (0.65–0.96)*	0.92 (0.84–1.00)
	Asian/Pacific Islander		0.67 (0.41–1.11)	1.10 (0.83–1.46)	0.87 (0.70–1.07)
	American Indian/Alaska Native		1.13 (0.64–2.01)	0.85 (0.57–1.29)	0.83 (0.64–1.07)
	Multiracial/Other		1.13 (0.77–1.68)	0.98 (0.75–1.26)	1.00 (0.89–1.12)
College degree vs. no college degree			1.07 (0.94–1.21)	1.10 (1.01–1.20)*	1.01 (0.94–1.08)
Income: vs. More than 2X poverty line					
	Below poverty line		0.74 (0.54–1.02)	0.82 (0.69–0.97)*	0.95 (0.88–1.04)
	Up to 2X poverty line		0.82 (0.68–0.98)*	0.90 (0.81–1.00)	0.97 (0.90–1.06)
Geographic area of residence: vs. Large metropolitan area					
	Small metropolitan area		0.97 (0.88–1.10)	1.07 (0.98–1.17)	1.08 (1.00–1.16)*
	Non-metropolitan area		0.91 (0.77–1.08)	1.14 (1.01–1.29)*	1.03 (0.93–1.14)
Residence in medical cannabis legal states vs. no legal states			0.94 (0.80–1.10)	0.77 (0.72–0.82)***	0.99 (0.93–1.05)
Chronic medical condition (M, SE)			1.11 (1.05–1.18)***	1.09 (1.05–1.14)***	1.05 (1.01–1.10)*
Past-year any mental illness: vs. None					
	Mild		1.40 (1.18–1.67)***	1.07 (0.95–1.22)	1.04 (0.96–1.13)
	Moderate		1.58 (1.28–1.94)***	1.00 (0.88–1.15)	1.12 (1.03–1.22)*
	Severe		1.87 (1.57–2.24)***	1.09 (0.96–1.23)	1.07 (0.97–1.17)
Nicotine dependence vs. No dependence			0.71 (0.55–0.92)*	0.51 (0.40–0.64)***	0.89 (0.81–0.99)*
Alcohol use disorder vs. No disorder			1.27 (1.04–1.54)*	0.68 (0.56–0.82)***	0.97 (0.91–1.03)
Past-year illicit drug use excluding cannabis vs. No use			1.62 (1.25–2.10)***	0.57 (0.45–0.73)***	1.16 (1.08–1.24)***
Great risk perception for using cannabis 1–2 times weekly vs. No great risk perception			0.51 (0.42–0.63)***	1.47 (1.35–1.61)***	1.12 (0.97–1.30)
CUD severity: vs. No CUD					1.06 (0.99–1.15)
		Mild			1.11 (1.01–1.12)*
		Moderate			1.12 (1.02–1.24)*
		Severe			
Model statistics			Sample N=34,287; Population N=197,455,984; Design df=50	Sample N=9033; Population N=45,654,793; Design df=50	Sample N=12,789; Population N=58,812,192; Design df=50

⁎ *p*<.05; **p<.01; ***p<.001

### 3.5 Correlates of CBD-only use versus cannabis-only use

The third column of Table 4 shows that compared to cannabis-only use, the likelihood of CBD-only use was significantly associated with middle and older ages (e.g., IRR=1.94, 95 % CI=1.70–2.22 for the 65+ age group), female gender (IRR=1.43, 95 % CI=1.31–1.57), college education (IRR=1.10, 95 % CI=1.01–1.20), non-metropolitan area residence (IRR=1.14, 95 % CI=1.01–1.29), chronic illness (IRR=1.09, 95 % CI=1.05–1.14), and great cannabis risk perception (1.47, 95 % CI=1.35–1.61). Being non-Hispanic Black or Hispanic, income below poverty, residence in medical cannabis legal states, nicotine dependence, alcohol use disorder, and illicit drug use were associated with a lower likelihood of CBD-only use.

### 3.6 Correlates of CBD-cannabis co-use versus cannabis-only use

The fourth column of Table 4 shows that compared to cannabis-only use, the likelihood of CBD-cannabis co-use was significantly associated with female gender (IRR=1.17, 95 % CI=1.10–1.25), small metropolitan area residence (IRR=1.08, 95 % CI=1.00–1.16), chronic illness (IRR=1.05, 95 % CI=1.01–1.10), moderate mental illness (IRR=1.12, 95 % CI=1.03–1.22), illicit drug use (IRR=1.16, 95 % CI=1.08–1.24), and moderate (IRR=1.11, 95 % CI=1.01–1.12) and severe (IRR=1.12, 95 % CI=1.02–1.24) CUD. Being non-Hispanic Black and nicotine dependence were associated with a lower likelihood of CBD-cannabis co-use.

## 4 Discussion

In 2022, one out of five U.S. adults reported having used CBD in the preceding year, which is close to the 23 % rate of cannabis use. The study also shows the high prevalence of CBD-cannabis co-use, as almost two thirds of CBD users also used cannabis and more than one half of cannabis users also used CBD. With increasing cannabis legalization and use prevalence (Caulkins, 2024) and demand for CBD products (Grand View Research., 2024), co-use of CBD and cannabis is projected to grow rapidly.

Consistent with the findings of previous studies of young adults (e.g., Fedorova et al., 2021), multivariable findings show that CBD use, with or without cannabis use, was consistently higher among women but lower among Black and Hispanic individuals compared to non-Hispanic White individuals. The higher likelihood of CBD use among women may be because they are more likely to experience the kinds of health concerns and conditions (e.g., anxiety/depression, pain, and sleep problems) that CBD is often used for. Women also tend to use complementary and alternative medicine for chronic conditions more than do men (Alwhaibi and Sambamoorthi, 2016). The wellness industry, including CBD products, also often targets women more aggressively. Many CBD products are marketed for skincare, stress relief, and menstrual pain to appeal to women. The lower likelihood of CBD use among Blacks and Hispanics may be related to different cultural perceptions and attitudes towards cannabis and CBD among racial/ethnic groups. Access to CBD products can also vary significantly based on socioeconomic and geographic factors. CBD products can be expensive, and economic barriers may limit access for some racial/ethnic groups, especially among older adults with fixed income. Access to and use of CBD and other hemp-derived products versus THC products may also vary for those residing in states with or without medical and/or adult-use cannabis laws (Smart and Pacula, 2019).

As hypothesized, compared to non-users of CBD or cannabis, CBD-only users had more physical and mental health and substance use problems and lower cannabis risk perceptions. The significant associations between CBD-only use, compared to non-use of CBD or cannabis, and health problems show that despite largely uncertain medicinal effects of CBD (Kirkland et al., 2022, Revol et al., 2024), the users were likely attracted to perceived benefits or expectations of the benefits. As also hypothesized, the likelihood of CBD-only use, compared to cannabis-only use, was higher among older adults and those with higher SES and more chronic illnesses, but fewer substance use problems. As older adults had more physical health problems, it was expected that the likelihood of CBD-only use, compared to cannabis-only use, was significantly higher among older adults. However, CBD-only users and cannabis-only users did not differ on mental illness status, suggesting that mental illness is equally present in CBD-only and cannabis-only groups.

Some states approve use of medical cannabis, along with CBD, for some psychiatric problems (Scherma et al., 2020). Like CBD users, medical cannabis users perceived reduction in depression, anxiety, and stress (Cuttler et al., 2018). While these perceived mental health benefits may be significant reasons for people turning to cannabis and/or CBD use, nonmedical cannabis use was also found to impede recovery and mental healthcare use (Bahorik et al., 2018). Studies have also shown that compared to cannabis non-users, cannabis users and those with CUD have significantly higher odds of psychiatric and substance use disorders (Onaemo et al., 2021, Padwa et al., 2022) and that cannabis use, especially among youth and young adults, is a risk factor for mental disorders (Gobbi et al., 2019, Large et al., 2011, Urits et al., 2020). At the same time, CBD-only use versus cannabis-only use was also associated with higher cannabis risk perception. It appears that CBD-only users stayed away from cannabis use as they perceived THC use to be harmful but believed CBD to be therapeutic.

Compared to cannabis-only users, co-users of CBD and cannabis also had more mental as well as physical health problems. A study of young-adult cannabis and CBD users found that their CBD use was associated with health histories and motivations linked to pain and psychological problems (Fedorova et al., 2021). Our findings of age group not being a significant factor for co-use suggest that both young and older adult co-users shared similar motivations for using CBD. Our findings also show that the co-users are those at most risk in terms of potential harms of cannabis use, as they, compared to cannabis-only users, used cannabis at higher frequency, and were more likely to have moderate or severe CUD and illicit drug use problems. The co-users may also have believed that CBD could attenuate some of THC’s adverse effects (Moltke and Hindocha, 2021). However, previous controlled clinical laboratory studies and randomized clinical trials have shown that CBD can either attenuate or exacerbate the effects of THC by increasing the pharmacodynamics of THC (Arkell et al., 2019; Englund et al., 2013; Solowij et al., 2019; Zamarripa et al., 2023). While more research is needed to establish the safety and drug-drug interactions of CBD and cannabis co-use, the co-users with CUD and illicit drug use problems need help.

In the absence of research on drug-drug interactions and other safety controls, including pesticides and heavy metals (Gardener et al., 2022), the high rate of CBD use among those with physical and mental health problems is concerning as they are more likely to be on other medications. Research has shown that CBD has intrinsic pharmacologic effects and associated adverse drug events along with the potential for pharmacokinetic and pharmacodynamic drug-drug interactions (Balachandran et al., 2021; Brown & Winterstein, 2019). Again, more research is needed to examine how CBD affects the pharmacodynamics and pharmacokinetics of other substances, including psychotropic medications, cannabis, nicotine, alcohol, opioids, and illicit drugs, and its potential as a substitute for other substances.

The study has some limitations due to the NSDUH data constraints. First, the lack of data on types of CBD products and use reasons, frequency and dosage limited the study scope. Second, the NSDUH did not distinguish CBD from other hemp-derived products containing Δ^8^- or Δ^10^-THC that are widely available in the market (Geci et al., 2023). Public interest in Δ^8^-THC has been surging in recent years especially in states where Δ^9^-THC is restricted (Leas et al., 2022). Because cannabis plants contain only trace amounts of Δ^8^- or Δ^10^-THC, they are usually made synthetically from CBD and added to edible and inhaled products that are marketed as legal hemp products (Huang et al., 2024, Kaczor et al., 2024, Babalonis et al., 2021;). Third, given the low degree of consumer knowledge of THC and CBD levels (Hammond & Goodman, 2022), the reliability and validity of CBD use report is also a suspect. Fourth, since NSDUH is based on self-report data, the validity of respondents’ self-reported CBD, cannabis, and other substance use may have been affected by social desirability bias. Fifth, geographic differences in access to and use of CBD or hemp-derived and THC products were not captured.

Despite these limitations, the study findings expand the knowledge about CBD users by examining three groups—CBD-only users, cannabis-only users, and CBD-cannabis co-users—using the nationally representative U.S. adult sample. Implications of the findings are: First, more epidemiologic research is needed to examine demographic differences in CBD use patterns and reasons especially with increasing legalization and normalization of hemp-based and THC products. Second, special research and clinical attention needs to be paid to co-users of CBD and cannabis to discern potential benefits and negative effects of co-use. Third, those who used CBD for their mental health problems need to be provided information on the importance of seeking professional advice for mental health issues. Fourth, the high prevalence of self-reported CBD use especially among those with physical and mental health problems warrants public health warnings about potential side effects and drug interactions, especially with long-term use. Fifth, more effective laws and regulations are needed to ensure safety of hemp and THC consumer products. The existing laws are often confusing. For example, under the Federal Food, Drug, and Cosmetics Act, the FDA prohibits the addition of CBD, Δ^8^-, or Δ^10^-THC to food or beverage; however, CBD-containing dietary supplements are marketed using the structure/function claims (Li et al., 2021, Mead, 2019). The FDA acknowledged that the existing regulatory framework for foods and supplements are not appropriate for CBD (U.S. FDA, 2023). Policy development and reforms also need to be based on strong economic and social justice goals and in ways to protect science and public health from corporate influence (Sevigny et al., 2023; Shover & Humphreys, 2019).

## Research ethics

This study based on de-identified public-domain data was exempt by the University of Texas at Austin’s Institutional Review Board.

## Funding source

This study did not receive any funding.

## Author contributions

study conceptualization: NGC, BYC; data management: NGC; data analysis and interpretation: NGC, CNM; manuscript draft: NGC, BYC; final editing: NGC, BYC, CNM

## CRediT authorship contribution statement

**C. Nathan Marti:** Writing – review & editing, Methodology. **Bryan Y. Choi:** Writing – review & editing, Writing – original draft, Investigation, Conceptualization. **Namkee G Choi:** Conceptualization, Investigation, Methodology, Software, Data curation and Analysis, Writing- Original draft preparation and final editing.

## Declaration of Competing Interest

The authors declare that there is no conflict of interest.

## Data Availability

This study is based on de-identified public-domain data (The National Survey on Drug Use and Health).
